# A tetra­nuclear nickel(II) complex, [Ni_4_(*L*)_4_](ClO_4_)_4_·C_2_H_3_N·2H_2_O, with an asymmetric Ni_4_O_4_ open-cubane-like core

**DOI:** 10.1107/S2056989021012408

**Published:** 2022-01-14

**Authors:** R. N. Patel, S. K. Patel, A. K. Patel, N. Patel, Ray J. Butcher

**Affiliations:** a Department of Chemistry, APS University, Rewa 486003, India; b Department of Chemistry, Howard University, 525 College Street NW, Washington DC 20059, USA

**Keywords:** crystal structure, cubane structure, Schiff base complexes, nickel

## Abstract

A tetra­nuclear complex with an open-cubane-like core structure was synthesized from 2-meth­oxy-6-(pyridin-2-yl-hydrazonometh­yl)phenol (**HL**), namely, *cyclo*-tetra­kis­(μ-2-meth­oxy-6-{[2-(pyridin-2-yl)hydrazin-1-yl­idene]meth­yl}pheno­lato)tetra­nickel(II) tetra­kis­(perchlorate) aceto­nitrile monosolvate dihydrate, [Ni_4_(C_13_H_12_N_3_O_2_)_4_](ClO_4_)_4_·C_2_H_3_N·2H_2_O, and characterized using micro-analytical and spectroscopic techniques. The crystal-structure determination reveals the formation of a distorted Ni_4_O_4_ cubane-like core architecture encapsulated by four hydrazone Schiff base (**HL**) mol­ecules. A open-cube tetra­nuclear architecture is created in which nickel(II) ions of the NiN_2_O_3_ unit are connected by μ_2_-O anions of the phenolate moiety of **HL**. In this complex, each Ni centre has a slightly distorted square-pyramidal coordination environment. The supra­molecular architectures are stabilized *via* the presence of various inter­molecular hydrogen bonds and (ar­yl–aryl, ar­yl–chelate and chelate–chelate) stacking inter­actions.

## Abstract 2

A tetra­nuclear complex with an open-cubane like structure was synthesized from 2-meth­oxy-6-(pyridin-2-yl-hydrazonometh­yl)-phenol and characterized using micro-analytical and spectroscopic techniques, and single-crystal X-ray diffraction analysis.

## Chemical context

Polynuclear metal(II) complexes have attracted much attention owing to their structural variety and significant applications in biology, catalysis, mol­ecular recognition and magnetism (Alcantara *et al.*, 2006 ▸; Powell, 2003 ▸). As such, complexes containing a tetra­nuclear cubane-like core have been an important class of compounds (Yang *et al.*, 2005). The synthesis of such polynuclear metal complexes can often be promoted with the use of polydentate Schiff base ligands possessing nitro­gen and oxygen donor atoms. Such Schiff bases are known to form high nuclearity complexes with inter­esting architectures, and the hydroxyl groups and other donor atoms are often suitable for the synthesis of polynuclear complexes (Gungor & Kara, 2015 ▸; Dutta *et al.*, 2020 ▸; Shit *et al.*, 2013 ▸). Several tetra­nuclear nickel(II) complexes have also been synthesized and their different electronic properties explored (Lin *et al.*, 2011 ▸; Nihei *et al.*, 2003 ▸; Zhang *et al.*, 2012 ▸; Liu *et al.*, 2012 ▸; Shit *et al.*, 2013 ▸). As part of our study of polynuclear complexes, we have been inter­ested in cubane-like structures to build complexes with high nuclearity (Ray *et al.*, 2009 ▸; Chakraborty *et al.*, 2009 ▸; Sagar *et al.*, 2017 ▸; Pouralimardan *et al.*, 2007 ▸; Patel *et al.*, 2019 ▸). In this article, the results were obtained with the Schiff base ligand (**HL**) 2-meth­oxy-6-(pyridin-2-yl-hydrazonometh­yl)-phenol, which can bind one or two metal ions, simultaneously. The stoichiometric reaction of nickel(II) perchlorate hexa­hydrate with this ligand resulted the formation of Ni_4_O_4_ distorted cubane-like structure described herein. 
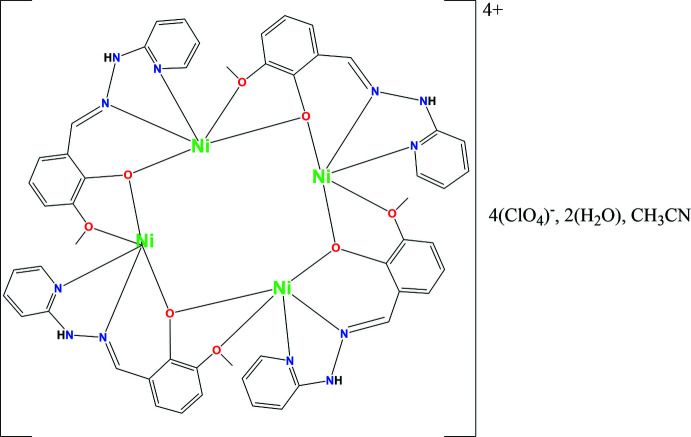


## Structural commentary

**Figure 1:**
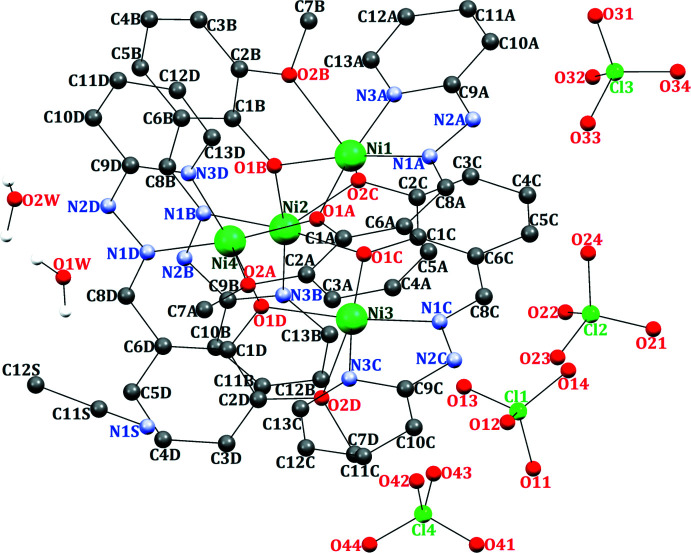
Mol­ecular structure of the tetra­nuclear nickel complex, [Ni_4_(*L*)_4_](ClO_4_)_4_·C_2_H_3_N·2H_2_O. Displacement ellipsoids are drawn at the 30% probability level.

**Figure 2:**
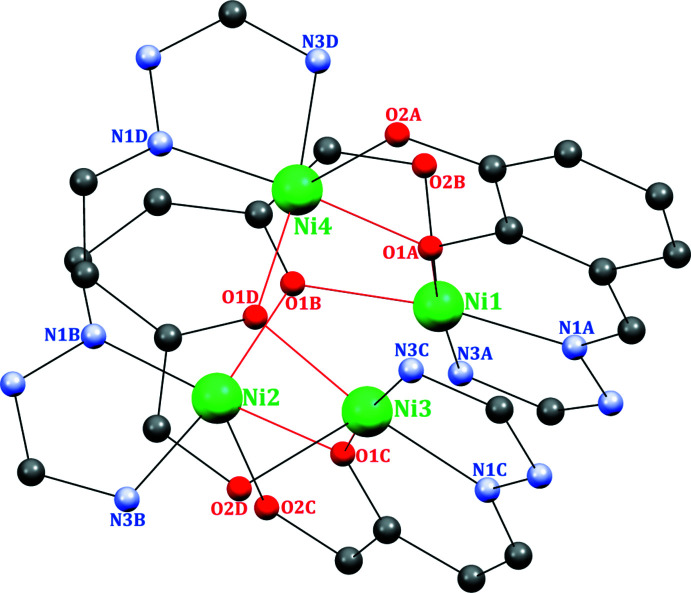
Ball-and-stick figure of the tetra­nuclear nickel complex, illustrating the coordination sphere about the nickel centres.

The hydrazone Schiff base (**HL**) was prepared by the reaction of 2-hydrazino­pyridine and 2-hy­droxy-3-meth­oxy­benz­alde­hyde in a 1:1 ratio in ethanol. The reaction of nickel perchlorate hexa­hydrate and the **HL** ligand yielded a tetra­nuclear open-cubane-like complex with an Ni_4_O_4_ core-type architecture. The tetra­nuclear complex is formulated as [Ni_4_(*L*)_4_](ClO_4_)_4_·C_2_H_3_N·2H_2_O (Fig. 1 ▸). Selected bond parameters are given in Table 1 ▸. The crystal-structure analysis reveals the formation of a distorted Ni_4_O_4_ cubane-like core. In this complex, four **HL** mol­ecules coordinate to the four nickel centres as a penta­dentate ligand (Fig. 2 ▸). The deprotonated Schiff base (**L^−^**) ligand coordinates in a penta­dentate mode (μ_2_-O_phenolate_, η^1^-N_imino_, η^1^-N_pyridin_, η^1^-O_meth­oxy_), thus forming eight fused metal chelate rings (four five-membered and four six-membered rings). Such a coordination pattern results in a distorted square-pyramidal coordination sphere around each nickel(II) ion. The distortion in the square-pyramidal geometry is shown by the τ index (τ_5_, with values of 0 for a perfect square pyramid and 1 for a perfect trigonal bipyramid; Addison *et al.*, 1984 ▸). The values for each Ni^II^ ion are 0.0383 for Ni1, 0.0050 for Ni2, 0.0033 for Ni3 and 0.0250 for Ni4. The fact that the τ-values are very close to zero indicates that the geometries around each Ni centre are slightly distorted from a perfect square-pyramidal environment.

The hydroxyl group of each **HL** phenol is deprotonated and the oxygen atoms bridge two nickel centres. Similarly, the oxygen atom of the meth­oxy group coordinates to a second nickel centre in a μ_2_-mode. Each nickel centre is connected to the μ_2_-oxygen atoms, resulting in the construction of an Ni_4_O_4_ cubane-like core (Fig. 2 ▸). The basal plane of each nickel centre is constituted by one phen­oxy oxygen, one meth­oxy oxygen, one azomethine nitro­gen and one pyridine nitro­gen atom. As a result of its weakly coordinating nature, each meth­oxy oxygen remains in an axial position. The Ni—N/O bond lengths are in the range 1.932 (7)–1.988 (5) Å and are very close to these reported for similar tetra­nuclear cubane-core-type complexes (Zhang *et al.*, 2011 ▸, 2013 ▸; Yu *et al.*, 2011 ▸; Tong *et al.*, 2002 ▸; Mandal *et al.*, 2008 ▸; Clemente-Juan *et al.*, 2000 ▸; Li *et al.*, 2006 ▸; Sun *et al.*, 2011 ▸; Saha *et al.*, 2014 ▸; Yang *et al.*, 2006 ▸).

## Supra­molecular features

**Figure 3:**
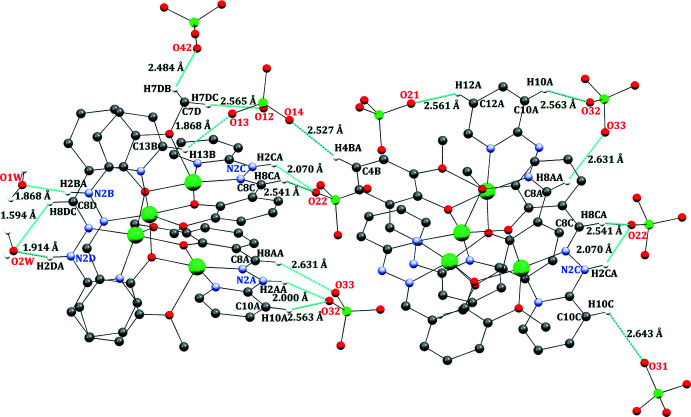
Inter­molecular N—H⋯O and C—H⋯O hydrogen bonding (drawn as dotted lines) between the tetra­nuclear complex, perchlorate counter-ions and water mol­ecules of crystallization.

**Figure 4:**
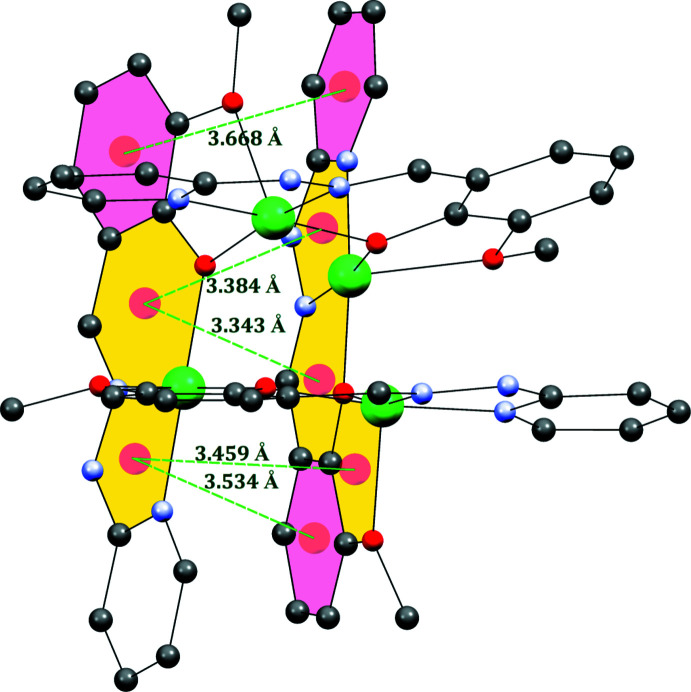
Diagram showing the π–π stacking inter­actions (drawn as dashed lines) observed in the complex.

**Figure 5:**
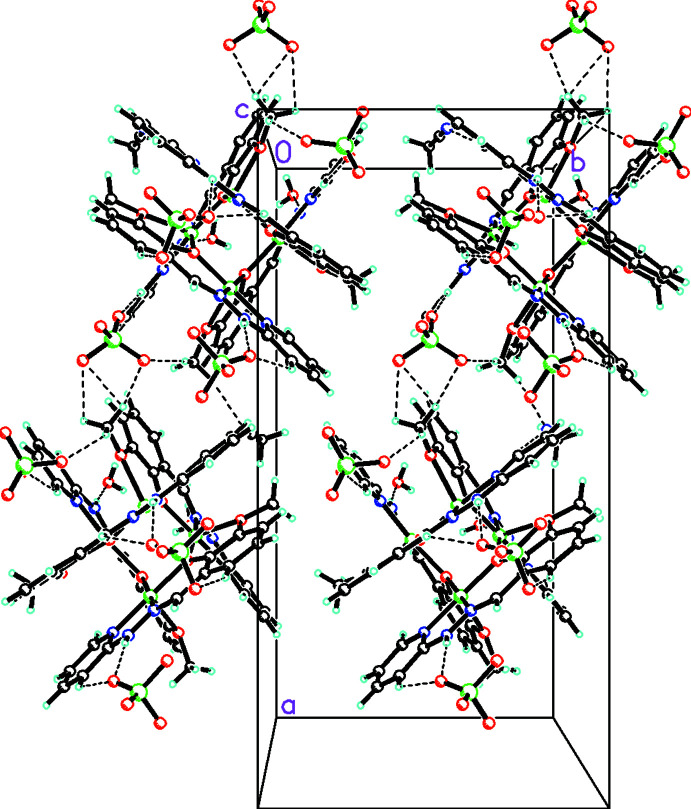
Crystal packing diagram viewed along *a*-axis of the complex.

In the polynuclear crystal, inter­molecular hydrogen-bonding inter­actions are detected involving C—H and N—H donors from the hydrazone Schiff base and acceptor oxygen atoms of perchlorate counter-ions and solvate water mol­ecules (Fig. 3 ▸). The important hydrogen-bonding parameters are collected in Table 2 ▸. The two tetra­nuclear complexes are inter­connected through inter­molecular hydrogen bonding between C—H⋯O and N—H⋯O hydrogen bonds with the perchlorate ions, forming heterosynthons (Fig. 3 ▸). Additionally, oxygen atoms of solvate water mol­ecules also act as acceptor atoms for inter­molecular hydrogen bonds. Furthermore, stabilization of the tetra­nuclear crystal lattice is facilitated by the presence of various weak (ar­yl–aryl, ar­yl–chelate and chelate–chelate) intra­molecular stacking inter­actions (Fig. 4 ▸). The ortho­rhom­bic cell contains four formula units, and the packing is shown in Fig. 5 ▸. The entire stacking pattern reveals that the inter­molecular hydrogen bonds remain between perchlorate counter-ions and C–H/N–H moieties of the same mol­ecule or adjacent mol­ecules. Similarly, solvate water mol­ecules also exert cooperative inter­molecular hydrogen bonds from C—H/N—H moieties of the complex, and the crystal lattice is also stabilized *via* π–π stacking inter­actions [centroid–centroid distances = 3.343 (3)–3.668 (3) Å].

## Database survey

A search of the Cambridge Structural Database (CSD; Groom *et al.*, 2016 ▸) for 2-meth­oxy-6-(pyridin-2-yl-hydrazonometh­yl)phenol gave no results. Several tetra­nuclear nickel complexes have been synthesized with several Schiff base ligands (Lin *et al.*, 2011 ▸; Liu *et al.*, 2012 ▸; Nihei *et al.*, 2003 ▸; Saha *et al.*, 2014 ▸; Shit *et al.*, 2013 ▸; Zhang *et al.*, 2012 ▸).

## Synthesis and crystallization

A mixture of 2-hydrazino­pyridine (0.327 g, 3.0 mmol) and 2-hy­droxy-3-meth­oxy­benzaldehyde (0.456 g, 3.0 mmol) in 30 mL of ethanol was refluxed for 3 h. The resulting light-yellow solution was cooled to room temperature. The obtained crystalline material was filtered off, washed with ethanol and kept in a CaCl_2_ desiccator. Yield 80%. Analysis calculated for C_13_H_13_N_3_O_2_: C, 64.18; H, 5.38; N, 17.27%. Found: C, 64.11; H, 5.27; N, 17.18%. FTIR (KBr cm^−1^): 1648, for (>C=N) and 3480 (–OH). The tetra­nuclear nickel complex was synthesized by taking an equimolar methano­lic solution (10 ml) of the **HL** ligand (0.243 g, 1.0 mmol) and nickel perchlorate hexa­hydrate (0.365 g, 1.0 mmol). The resulting solution was stirred for 3 h. The obtained green crystals, suitable for diffraction studies, were filtered off and washed with cold methanol and kept in a CaCl_2_ desiccator. Yield 62%. Analysis calculated for C_54_H_55_Cl_4_N_13_Ni_4_O_26_: C, 38.63; H, 3.30; N, 10.84%. Found: C, 38.28; H, 3.28; N, 10.98%. FTIR (KBr, υ, cm^−1^): 1626 (>C=N), 1537 (C—O), 487 (Ni—O) and 421 (Ni—N).

## Refinement

Crystallographic data and refinement details are presented in Table 3 ▸. H atoms were located in difference-Fourier maps and constrained to ride on their parent atoms with with C—H bond distances of 0.95 Å (aromatic H), 0.98 Å (methyl H) and 0.88 Å (N—H) and were refined as riding with isotropic displacement parameters 1.2 and 1.5 times those of the parent C/N atoms. Water H atoms were refined isotropically with *U*ĩso(H) = 1.5*U*eq(O). Three of the four perchlorate anions are disordered over two orientations and were restrained to have tetra­hedral geometries with occupancies of 0.57 (6)/0.43 (6), 0.412 (13)/0.488 (13), and 0.806 (12)/0.194 (12), respectively.

## Supplementary Material

Crystal structure: contains datablock(s) I. DOI: 10.1107/S2056989021012408/yy2002sup1.cif

Structure factors: contains datablock(s) I. DOI: 10.1107/S2056989021012408/yy2002Isup2.hkl

CCDC reference: 2096266

Additional supporting information:  crystallographic information; 3D view; checkCIF report

## Figures and Tables

**Table 1:** Selected geometric parameters (Å, °)

Ni1—N1*A*	1.932 (7)	Ni3—N1*C*	1.932 (7)
Ni1—O1*A*	1.960 (5)	Ni3—O1*C*	1.965 (5)
Ni1—N3*A*	1.980 (6)	Ni3—N3*C*	1.975 (6)
Ni1—O1*B*	1.998 (5)	Ni3—O1*D*	1.993 (5)
Ni1—O2*B*	2.276 (4)	Ni3—O2*D*	2.264 (5)
Ni2—N1*B*	1.935 (6)	Ni4—N1*D*	1.948 (7)
Ni2—O1*B*	1.962 (4)	Ni4—O1*D*	1.950 (5)
Ni2—N3*B*	1.971 (6)	Ni4—N3*D*	1.969 (6)
Ni2—O1*C*	1.997 (5)	Ni4—O1*A*	1.993 (5)
Ni2—O2*C*	2.257 (5)	Ni4—O2*A*	2.283 (5)
			
N1*A*—Ni1—O1*A*	92.2 (2)	N1*C*—Ni3—O1*C*	91.2 (2)
N1*A*—Ni1—N3*A*	81.6 (2)	N1*C*—Ni3—N3*C*	82.4 (3)
O1*A*—Ni1—N3*A*	173.5 (2)	O1*C*—Ni3—N3*C*	172.3 (2)
N1*A*—Ni1—O1*B*	171.2 (2)	N1*C*—Ni3—O1*D*	172.1 (2)
O1*A*—Ni1—O1*B*	87.1 (2)	O1*C*—Ni3—O1*D*	88.1 (2)
N3*A*—Ni1—O1*B*	98.8 (2)	N3*C*—Ni3—O1*D*	97.7 (2)
N1*A*—Ni1—O2*B*	113.4 (2)	N1*C*—Ni3—O2*D*	111.5 (2)
O1*A*—Ni1—O2*B*	98.83 (19)	O1*C*—Ni3—O2*D*	99.81 (19)
N3*A*—Ni1—O2*B*	85.3 (2)	N3*C*—Ni3—O2*D*	86.5 (2)
O1*B*—Ni1—O2*B*	75.39 (17)	O1*D*—Ni3—O2*D*	76.3 (2)
N1*B*—Ni2—O1*B*	91.3 (2)	N1*D*—Ni4—O1*D*	89.9 (2)
N1*B*—Ni2—N3*B*	82.0 (2)	N1*D*—Ni4—N3*D*	81.9 (3)
O1*B*—Ni2—N3*B*	171.8 (2)	O1*D*—Ni4—N3*D*	170.6 (2)
N1*B*—Ni2—O1*C*	172.1 (2)	N1*D*—Ni4—O1*A*	169.1 (2)
O1*B*—Ni2—O1*C*	88.8 (2)	O1*D*—Ni4—O1*A*	89.5 (2)
N3*B*—Ni2—O1*C*	97.1 (2)	N3*D*—Ni4—O1*A*	97.8 (2)
N1*B*—Ni2—O2*C*	111.7 (2)	N1*D*—Ni4—O2*A*	116.3 (2)
O1*B*—Ni2—O2*C*	97.69 (18)	O1*D*—Ni4—O2*A*	100.3 (2)
N3*B*—Ni2—O2*C*	89.2 (2)	N3*D*—Ni4—O2*A*	87.4 (2)
O1*C*—Ni2—O2*C*	76.02 (19)	O1*A*—Ni4—O2*A*	74.5 (2)

**Table 2:** Hydrogen-bond geometry (Å, °)

*D*—H⋯*A*	*D*—H	H⋯*A*	*D*⋯*A*	*D*—H⋯*A*
C7*B*—H7*BB*⋯N1*S* ^i^	0.98	2.60	3.523 (11)	157
C13*B*—H13*B*⋯O13	0.95	2.42	3.126 (9)	131
C13*C*—H13*C*⋯N1*S*	0.95	2.59	3.348 (11)	137
N2*D*—H2*DA*⋯O2*W*	0.88	1.91	2.720 (9)	152
C7*D*—H7*DC*⋯O12	0.98	2.56	3.389 (10)	142
C12*S*—H12*G*⋯O14^ii^	0.98	2.37	3.335 (12)	168
O2*W*—H2*W*1⋯O11^ii^	0.84 (3)	2.12 (7)	2.795 (8)	138 (9)
O2*W*—H2*W*2⋯Cl4^ii^	0.83 (3)	2.78 (3)	3.599 (6)	170 (9)

Symmetry codes: (i) x+{\script{1\over 2}}, -y+{\script{3\over 2}}, z; (ii) -x+1, -y+1, z-{\script{1\over 2}}.

**Table 3:** Experimental details

Crystal data	
Chemical formula	[Ni_4_(C_13_H_12_N_3_O_2_)_4_](ClO_4_)_4_·C_2_H_3_N·2H_2_O
*M*_r_	1678.75
Crystal system, space group	Orthorhombic, *P* *n* *a*2_1_
Temperature (K)	100
*a*, *b*, *c* (Å)	23.5976 (6), 11.8723 (3), 22.2989 (6)
*V* (Å^3^)	6247.2 (3)
*Z*	4
Radiation type	Mo *K*α
μ (mm^−1^)	1.46
Crystal size (mm)	0.25 × 0.11 × 0.09

Data collection	
Diffractometer	Bruker APEXII CCD
Absorption correction	Multi-scan (*SADABS*; Krause *et al.*, 2015 ▸)
*T*_min_, *T*_max_	0.554, 0.765
No. of measured, independent and observed [*I* > 2σ(*I*)] reflections	86241, 14617, 11629
*R*_int_	0.068
(sin θ/λ)_max_ (Å^−1^)	0.667

Refinement	
*R*[*F* ^2^ > 2σ(*F* ^2^)], *wR*(*F* ^2^), *S*	0.048, 0.125, 1.06
No. of reflections	14617
No. of parameters	1051
No. of restraints	320
H-atom treatment	H atoms treated by a mixture of independent and constrained refinement
Δρ_max_, Δρ_min_ (e Å^−3^)	0.84, −1.30
Absolute structure	Flack *x* determined using 4529 quotients [(*I* ^+^)−(*I* ^−^)]/[(*I* ^+^)+(*I* ^−^)] (Parsons *et al.*, 2013 ▸)
Absolute structure parameter	0.024 (5)

Computer programs: *APEX2* (Bruker, 2005 ▸) *SAINT* (Bruker, 2002 ▸), *SHELXT* (Sheldrick 2015*a* ▸), *SHELXL2018/3* (Sheldrick, 2015*b* ▸) and *SHELXTL* (Sheldrick 2008 ▸).

## supplementary crystallographic information

### Crystal data

**Table:**

[Ni_4_(C_13_H_12_N_3_O_2_)_4_](ClO_4_)_4_·C_2_H_3_N·2H_2_O	*D*_x_ = 1.785 Mg m^−^^3^
*M**_r_* = 1678.75	Mo *K*α radiation, λ = 0.71073 Å
Orthorhombic, *P**n**a*2_1_	Cell parameters from 9461 reflections
*a* = 23.5976 (6) Å	θ = 2.4–26.3°
*b* = 11.8723 (3) Å	µ = 1.46 mm^−^^1^
*c* = 22.2989 (6) Å	*T* = 100 K
*V* = 6247.2 (3) Å^3^	Needle, green
*Z* = 4	0.25 × 0.11 × 0.09 mm
*F*(000) = 3432	

### Data collection

**Table:**

Bruker APEXII CCD diffractometer	11629 reflections with *I* > 2σ(*I*)
φ and ω scans	*R*_int_ = 0.068
Absorption correction: multi-scan (SADABS; Krause *et al.*, 2015)	θ_max_ = 28.3°, θ_min_ = 1.9°
*T*_min_ = 0.554, *T*_max_ = 0.765	*h* = −31→31
86241 measured reflections	*k* = −15→15
14617 independent reflections	*l* = −26→29

### Refinement

**Table:**

Refinement on *F*^2^	Hydrogen site location: mixed
Least-squares matrix: full	H atoms treated by a mixture of independent and constrained refinement
*R*[*F*^2^ > 2σ(*F*^2^)] = 0.048	*w* = 1/[σ^2^(*F*_o_^2^) + (0.0571*P*)^2^ + 9.1783*P*] where *P* = (*F*_o_^2^ + 2*F*_c_^2^)/3
*wR*(*F*^2^) = 0.125	(Δ/σ)_max_ = 0.001
*S* = 1.06	Δρ_max_ = 0.84 e Å^−^^3^
14617 reflections	Δρ_min_ = −1.30 e Å^−^^3^
1051 parameters	Extinction correction: SHELXL-2018/3 (Sheldrick 2018), Fc^*^=kFc[1+0.001xFc^2^λ^3^/sin(2θ)]^-1/4^
320 restraints	Extinction coefficient: 0.00135 (19)
Primary atom site location: structure-invariant direct methods	Absolute structure: Flack *x* determined using 4529 quotients [(*I*^+^)-(*I*^-^)]/[(*I*^+^)+(*I*^-^)] (Parsons *et al.*, 2013)
Secondary atom site location: difference Fourier map	Absolute structure parameter: 0.024 (5)

### Special details

**Table:**

Geometry. All esds (except the esd in the dihedral angle between two l.s. planes) are estimated using the full covariance matrix. The cell esds are taken into account individually in the estimation of esds in distances, angles and torsion angles; correlations between esds in cell parameters are only used when they are defined by crystal symmetry. An approximate (isotropic) treatment of cell esds is used for estimating esds involving l.s. planes.

### Fractional atomic coordinates and isotropic or equivalent isotropic displacement parameters (Å^2^)

**Table:**

	*x*	*y*	*z*	*U*_iso_*/*U*_eq_	Occ. (<1)
Ni1	0.73671 (3)	0.62167 (6)	0.50776 (4)	0.01973 (18)	
Ni2	0.65087 (3)	0.46794 (6)	0.42646 (4)	0.01955 (17)	
Ni3	0.58915 (3)	0.60905 (7)	0.53570 (4)	0.02116 (18)	
Ni4	0.64341 (3)	0.75702 (6)	0.42586 (4)	0.02228 (18)	
O1A	0.6789 (2)	0.7401 (4)	0.5065 (2)	0.0261 (10)	
O2A	0.6144 (2)	0.9102 (4)	0.4796 (3)	0.0319 (12)	
N1A	0.7519 (2)	0.6456 (5)	0.5919 (3)	0.0282 (13)	
N2A	0.7897 (3)	0.5687 (5)	0.6153 (3)	0.0315 (13)	
H2AA	0.797624	0.565969	0.653817	0.06 (3)*	
N3A	0.7945 (2)	0.5026 (5)	0.5191 (3)	0.0251 (12)	
C1A	0.6699 (3)	0.8175 (6)	0.5507 (3)	0.0259 (15)	
C2A	0.6349 (3)	0.9109 (5)	0.5375 (4)	0.0284 (15)	
C3A	0.6212 (4)	0.9884 (6)	0.5812 (4)	0.0383 (19)	
H3AA	0.597016	1.049895	0.571891	0.046*	
C4A	0.6423 (4)	0.9769 (7)	0.6381 (4)	0.045 (2)	
H4AA	0.632679	1.030502	0.668040	0.054*	
C5A	0.6775 (4)	0.8881 (7)	0.6523 (4)	0.0406 (19)	
H5AA	0.692022	0.881058	0.691864	0.049*	
C6A	0.6920 (3)	0.8081 (6)	0.6086 (3)	0.0302 (16)	
C7A	0.5891 (4)	1.0134 (6)	0.4589 (4)	0.044 (2)	
H7AA	0.612658	1.077293	0.471255	0.066*	
H7AB	0.551164	1.021250	0.476297	0.066*	
H7AC	0.586234	1.011904	0.415072	0.066*	
C8A	0.7308 (3)	0.7216 (6)	0.6278 (3)	0.0311 (16)	
H8AA	0.741612	0.719868	0.668851	0.037*	
C9A	0.8139 (3)	0.4970 (6)	0.5750 (3)	0.0265 (15)	
C10A	0.8565 (3)	0.4202 (7)	0.5921 (4)	0.0326 (17)	
H10A	0.869548	0.417446	0.632430	0.039*	
C11A	0.8782 (3)	0.3517 (7)	0.5509 (4)	0.0363 (18)	
H11A	0.906120	0.298033	0.562076	0.044*	
C12A	0.8601 (3)	0.3578 (6)	0.4908 (4)	0.0342 (17)	
H12A	0.876312	0.310984	0.460768	0.041*	
C13A	0.8179 (3)	0.4346 (6)	0.4770 (4)	0.0298 (15)	
H13A	0.804979	0.439766	0.436785	0.036*	
O1B	0.71137 (17)	0.5813 (3)	0.4250 (2)	0.0231 (9)	
O2B	0.80171 (19)	0.7036 (4)	0.4463 (2)	0.0256 (10)	
N1B	0.6495 (2)	0.4613 (5)	0.3398 (3)	0.0254 (13)	
N2B	0.6028 (3)	0.4046 (5)	0.3185 (3)	0.0301 (13)	
H2BA	0.594259	0.403529	0.280135	0.07 (4)*	
N3B	0.5861 (2)	0.3646 (4)	0.4175 (3)	0.0252 (12)	
C1B	0.7471 (3)	0.6010 (5)	0.3779 (3)	0.0239 (14)	
C2B	0.7959 (3)	0.6656 (5)	0.3881 (3)	0.0240 (14)	
C3B	0.8342 (3)	0.6893 (6)	0.3426 (3)	0.0272 (15)	
H3BA	0.867365	0.732216	0.350462	0.033*	
C4B	0.8230 (3)	0.6488 (6)	0.2849 (3)	0.0302 (15)	
H4BA	0.849159	0.663339	0.253437	0.036*	
C5B	0.7750 (3)	0.5886 (6)	0.2734 (3)	0.0282 (15)	
H5BA	0.767612	0.563818	0.233670	0.034*	
C6B	0.7361 (3)	0.5626 (6)	0.3195 (3)	0.0252 (14)	
C7B	0.8561 (3)	0.7453 (7)	0.4645 (4)	0.0367 (18)	
H7BA	0.865425	0.812932	0.441373	0.055*	
H7BB	0.884944	0.687402	0.457407	0.055*	
H7BC	0.855123	0.763956	0.507341	0.055*	
C8B	0.6866 (3)	0.4975 (6)	0.3018 (3)	0.0260 (14)	
H8BA	0.681397	0.480901	0.260520	0.031*	
C9B	0.5706 (3)	0.3504 (6)	0.3597 (4)	0.0282 (15)	
C10B	0.5229 (3)	0.2854 (6)	0.3442 (4)	0.0352 (17)	
H10B	0.511292	0.278466	0.303583	0.042*	
C11B	0.4938 (3)	0.2330 (6)	0.3886 (4)	0.0369 (19)	
H11B	0.461388	0.188949	0.379192	0.044*	
C12B	0.5115 (3)	0.2435 (6)	0.4488 (4)	0.0316 (16)	
H12B	0.492177	0.204730	0.479900	0.038*	
C13B	0.5568 (3)	0.3103 (5)	0.4614 (4)	0.0298 (16)	
H13B	0.568312	0.319200	0.501973	0.036*	
O1C	0.64418 (19)	0.4911 (4)	0.5149 (2)	0.0252 (10)	
O2C	0.7018 (2)	0.3254 (4)	0.4662 (2)	0.0303 (11)	
N1C	0.6018 (2)	0.5844 (5)	0.6203 (3)	0.0281 (13)	
N2C	0.5735 (3)	0.6611 (5)	0.6559 (3)	0.0327 (14)	
H2CA	0.575273	0.658689	0.695341	0.14 (6)*	
N3C	0.5404 (2)	0.7317 (5)	0.5660 (3)	0.0270 (12)	
C1C	0.6662 (3)	0.4124 (6)	0.5522 (3)	0.0253 (15)	
C2C	0.6976 (3)	0.3228 (6)	0.5273 (4)	0.0299 (16)	
C3C	0.7222 (3)	0.2412 (6)	0.5633 (4)	0.0367 (18)	
H3CA	0.742965	0.181040	0.545793	0.044*	
C4C	0.7164 (3)	0.2480 (7)	0.6254 (4)	0.0385 (19)	
H4CA	0.733433	0.192783	0.650417	0.046*	
C5C	0.6862 (3)	0.3341 (7)	0.6501 (4)	0.0364 (18)	
H5CA	0.682536	0.337817	0.692492	0.044*	
C6C	0.6602 (3)	0.4172 (6)	0.6149 (4)	0.0290 (15)	
C7C	0.7241 (3)	0.2280 (6)	0.4358 (4)	0.041 (2)	
H7CA	0.764599	0.220712	0.444534	0.062*	
H7CB	0.718647	0.236542	0.392429	0.062*	
H7CC	0.704131	0.160439	0.449638	0.062*	
C8C	0.6291 (3)	0.5033 (6)	0.6458 (4)	0.0329 (17)	
H8CA	0.628574	0.500392	0.688326	0.040*	
C9C	0.5425 (3)	0.7411 (6)	0.6262 (4)	0.0315 (16)	
C10C	0.5134 (3)	0.8263 (7)	0.6577 (4)	0.0387 (18)	
H10C	0.516037	0.832498	0.700031	0.046*	
C11C	0.4814 (4)	0.8989 (7)	0.6252 (5)	0.047 (2)	
H11C	0.461404	0.957613	0.644892	0.056*	
C12C	0.4776 (4)	0.8880 (7)	0.5629 (4)	0.0383 (18)	
H12C	0.454778	0.938126	0.540173	0.046*	
C13C	0.5076 (3)	0.8034 (6)	0.5351 (4)	0.0314 (15)	
H13C	0.504959	0.795620	0.492831	0.038*	
O1D	0.5854 (2)	0.6484 (4)	0.4489 (2)	0.0273 (11)	
O2D	0.5083 (2)	0.5253 (4)	0.5041 (3)	0.0335 (11)	
N1D	0.6176 (3)	0.7490 (5)	0.3430 (3)	0.0319 (14)	
N2D	0.6544 (3)	0.7994 (6)	0.3044 (3)	0.0354 (15)	
H2DA	0.650230	0.794969	0.265219	0.06 (3)*	
N3D	0.7016 (3)	0.8547 (5)	0.3897 (3)	0.0287 (13)	
C1D	0.5384 (3)	0.6181 (5)	0.4174 (4)	0.0278 (15)	
C2D	0.4963 (3)	0.5530 (5)	0.4450 (3)	0.0276 (15)	
C3D	0.4487 (3)	0.5165 (6)	0.4145 (4)	0.0330 (17)	
H3DA	0.420861	0.472560	0.434478	0.040*	
C4D	0.4418 (3)	0.5441 (7)	0.3551 (4)	0.0381 (18)	
H4DA	0.408760	0.520128	0.334346	0.046*	
C5D	0.4820 (3)	0.6059 (6)	0.3257 (4)	0.0365 (18)	
H5DA	0.477119	0.623155	0.284384	0.044*	
C6D	0.5309 (3)	0.6444 (6)	0.3562 (4)	0.0319 (16)	
C7D	0.4621 (4)	0.4883 (7)	0.5406 (4)	0.045 (2)	
H7DA	0.451192	0.411766	0.528798	0.067*	
H7DB	0.429829	0.539144	0.535135	0.067*	
H7DC	0.473691	0.488523	0.582776	0.067*	
C8D	0.5718 (3)	0.7045 (6)	0.3210 (4)	0.0324 (16)	
H8DA	0.564968	0.712128	0.279187	0.039*	
C9D	0.6979 (3)	0.8568 (6)	0.3297 (4)	0.0333 (17)	
C10D	0.7367 (4)	0.9166 (7)	0.2944 (4)	0.0397 (19)	
H10D	0.734936	0.914895	0.251865	0.048*	
C11D	0.7779 (4)	0.9786 (7)	0.3244 (5)	0.046 (2)	
H11D	0.804852	1.020685	0.302010	0.056*	
C12D	0.7801 (3)	0.9795 (6)	0.3863 (5)	0.042 (2)	
H12D	0.807932	1.023129	0.406559	0.050*	
C13D	0.7415 (3)	0.9164 (6)	0.4182 (4)	0.0342 (17)	
H13D	0.742885	0.916202	0.460822	0.041*	
Cl1	0.52002 (8)	0.25270 (17)	0.65520 (9)	0.0359 (4)	
O11	0.4683 (2)	0.1969 (6)	0.6705 (3)	0.0526 (16)	
O12	0.5136 (3)	0.3696 (5)	0.6678 (3)	0.0580 (18)	
O13	0.5308 (4)	0.2377 (7)	0.5934 (3)	0.071 (2)	
O14	0.5644 (3)	0.2096 (7)	0.6908 (3)	0.072 (2)	
Cl2	0.64516 (10)	0.75422 (18)	0.80261 (10)	0.0462 (5)	
O21	0.6408 (13)	0.711 (2)	0.8612 (9)	0.044 (6)	0.57 (6)
O22	0.6361 (18)	0.6641 (19)	0.7612 (13)	0.072 (8)	0.57 (6)
O23	0.6043 (10)	0.8369 (17)	0.7913 (11)	0.056 (6)	0.57 (6)
O24	0.7018 (7)	0.797 (3)	0.7964 (16)	0.097 (9)	0.57 (6)
O21A	0.6366 (17)	0.693 (3)	0.8554 (11)	0.053 (11)	0.43 (6)
O22A	0.6610 (18)	0.677 (2)	0.7558 (11)	0.053 (8)	0.43 (6)
O23A	0.5915 (12)	0.800 (5)	0.7822 (16)	0.081 (11)	0.43 (6)
O24A	0.685 (2)	0.838 (3)	0.8092 (13)	0.093 (12)	0.43 (6)
Cl3	0.86202 (15)	0.6242 (4)	0.76039 (17)	0.0898 (11)	
O31	0.9121 (5)	0.6848 (11)	0.7412 (6)	0.144 (5)	
O32	0.8498 (7)	0.5395 (12)	0.7231 (6)	0.064 (4)	0.512 (13)
O33	0.8143 (6)	0.7097 (13)	0.7581 (8)	0.074 (4)	0.512 (13)
O34	0.8732 (11)	0.598 (2)	0.8193 (7)	0.076 (5)	0.512 (13)
O32A	0.8836 (6)	0.4985 (11)	0.7347 (6)	0.057 (4)	0.488 (13)
O33A	0.8224 (7)	0.6441 (15)	0.7248 (7)	0.079 (4)	0.488 (13)
O34A	0.8626 (12)	0.610 (2)	0.8193 (7)	0.068 (5)	0.488 (13)
Cl4	0.32147 (12)	0.5162 (3)	0.59552 (15)	0.0719 (8)	
O41	0.3017 (5)	0.4882 (10)	0.6510 (4)	0.076 (3)	0.806 (12)
O42	0.3541 (5)	0.6162 (7)	0.6010 (5)	0.063 (3)	0.806 (12)
O43	0.3553 (5)	0.4174 (9)	0.5809 (6)	0.092 (4)	0.806 (12)
O44	0.2846 (5)	0.5263 (11)	0.5485 (4)	0.087 (4)	0.806 (12)
O41A	0.2808 (14)	0.491 (4)	0.6390 (16)	0.099 (14)	0.194 (12)
O42A	0.3754 (9)	0.486 (4)	0.6243 (13)	0.106 (9)	0.194 (12)
O43A	0.3244 (17)	0.6325 (18)	0.5851 (18)	0.061 (8)	0.194 (12)
O44A	0.3147 (16)	0.457 (3)	0.5430 (13)	0.072 (9)	0.194 (12)
N1S	0.4678 (4)	0.9156 (9)	0.4038 (4)	0.075 (3)	
C11S	0.4759 (4)	0.9363 (7)	0.3557 (5)	0.051 (2)	
C12S	0.4883 (6)	0.9668 (10)	0.2935 (5)	0.071 (3)	
H12E	0.529373	0.972979	0.288126	0.106*	
H12F	0.470415	1.039174	0.284150	0.106*	
H12G	0.473352	0.908563	0.266681	0.106*	
O1W	0.5643 (5)	0.4311 (10)	0.2040 (5)	0.040 (4)	0.51 (2)
H1W1	0.538 (4)	0.468 (11)	0.2161 (18)	0.060*	0.51 (2)
H1W2	0.587 (6)	0.474 (11)	0.189 (9)	0.060*	0.51 (2)
O1WA	0.5995 (10)	0.3463 (16)	0.1981 (8)	0.096 (9)	0.49 (2)
H1W3	0.602 (13)	0.411 (14)	0.19 (2)	0.144*	0.49 (2)
H1W4	0.565 (3)	0.33 (2)	0.199 (5)	0.144*	0.49 (2)
O2W	0.6389 (3)	0.7126 (6)	0.1929 (3)	0.0547 (17)	
H2W1	0.6042 (15)	0.712 (10)	0.185 (5)	0.082*	
H2W2	0.651 (4)	0.670 (9)	0.167 (4)	0.082*	

### Atomic displacement parameters (Å^2^)

**Table:**

	*U*^11^	*U*^22^	*U*^33^	*U*^12^	*U*^13^	*U*^23^
Ni1	0.0225 (4)	0.0185 (4)	0.0181 (4)	0.0000 (3)	0.0010 (3)	−0.0011 (3)
Ni2	0.0213 (3)	0.0167 (3)	0.0207 (4)	−0.0003 (3)	0.0006 (4)	0.0004 (4)
Ni3	0.0231 (4)	0.0197 (4)	0.0206 (4)	0.0018 (3)	0.0008 (3)	0.0027 (3)
Ni4	0.0266 (4)	0.0175 (4)	0.0227 (4)	0.0018 (3)	0.0022 (4)	0.0019 (4)
O1A	0.031 (2)	0.018 (2)	0.029 (3)	0.0000 (18)	0.005 (2)	−0.003 (2)
O2A	0.034 (3)	0.020 (2)	0.042 (3)	0.008 (2)	0.005 (2)	0.004 (2)
N1A	0.027 (3)	0.029 (3)	0.029 (3)	−0.004 (2)	0.002 (2)	0.000 (3)
N2A	0.037 (3)	0.032 (3)	0.026 (3)	0.002 (3)	−0.002 (3)	0.005 (3)
N3A	0.026 (3)	0.024 (3)	0.025 (3)	−0.002 (2)	0.003 (2)	0.002 (2)
C1A	0.025 (3)	0.020 (3)	0.033 (4)	−0.003 (3)	0.006 (3)	−0.004 (3)
C2A	0.030 (3)	0.018 (3)	0.037 (4)	−0.006 (3)	0.010 (3)	−0.003 (3)
C3A	0.045 (4)	0.025 (4)	0.045 (5)	−0.004 (3)	0.014 (4)	−0.009 (3)
C4A	0.050 (5)	0.034 (4)	0.052 (6)	−0.006 (4)	0.016 (4)	−0.019 (4)
C5A	0.049 (5)	0.036 (4)	0.038 (5)	−0.007 (4)	0.008 (4)	−0.011 (4)
C6A	0.033 (4)	0.028 (3)	0.030 (4)	−0.006 (3)	0.005 (3)	−0.006 (3)
C7A	0.043 (4)	0.028 (4)	0.061 (6)	0.009 (3)	0.010 (4)	0.015 (4)
C8A	0.039 (4)	0.031 (4)	0.024 (4)	−0.008 (3)	0.002 (3)	−0.005 (3)
C9A	0.022 (3)	0.028 (3)	0.030 (4)	−0.008 (3)	0.004 (3)	0.004 (3)
C10A	0.025 (3)	0.038 (4)	0.035 (4)	0.002 (3)	−0.003 (3)	0.013 (3)
C11A	0.029 (4)	0.038 (4)	0.041 (5)	0.000 (3)	−0.006 (3)	0.011 (3)
C12A	0.034 (4)	0.027 (4)	0.042 (5)	0.002 (3)	0.002 (3)	−0.001 (3)
C13A	0.030 (4)	0.029 (4)	0.030 (4)	0.000 (3)	−0.002 (3)	0.002 (3)
O1B	0.026 (2)	0.024 (2)	0.019 (2)	0.0011 (17)	0.002 (2)	0.000 (2)
O2B	0.025 (2)	0.025 (2)	0.026 (3)	−0.0070 (19)	0.0051 (19)	−0.0003 (19)
N1B	0.027 (3)	0.022 (3)	0.028 (3)	−0.001 (2)	0.002 (2)	−0.001 (2)
N2B	0.029 (3)	0.036 (3)	0.025 (4)	−0.006 (3)	−0.001 (2)	0.000 (3)
N3B	0.028 (3)	0.018 (2)	0.030 (3)	0.003 (2)	0.002 (2)	0.000 (2)
C1B	0.024 (3)	0.019 (3)	0.029 (4)	0.003 (2)	0.004 (3)	−0.001 (3)
C2B	0.025 (3)	0.020 (3)	0.027 (4)	0.001 (3)	0.006 (3)	−0.002 (3)
C3B	0.029 (3)	0.029 (4)	0.024 (4)	0.000 (3)	0.005 (3)	−0.002 (3)
C4B	0.032 (4)	0.036 (4)	0.023 (4)	−0.001 (3)	0.010 (3)	0.003 (3)
C5B	0.035 (4)	0.029 (3)	0.020 (4)	−0.001 (3)	0.002 (3)	−0.004 (3)
C6B	0.031 (3)	0.021 (3)	0.024 (4)	0.000 (3)	0.003 (3)	0.002 (3)
C7B	0.029 (4)	0.043 (4)	0.038 (5)	−0.010 (3)	0.000 (3)	−0.010 (4)
C8B	0.031 (3)	0.023 (3)	0.025 (4)	0.003 (3)	0.003 (3)	−0.005 (3)
C9B	0.029 (3)	0.021 (3)	0.034 (4)	0.001 (3)	−0.001 (3)	−0.002 (3)
C10B	0.034 (4)	0.032 (4)	0.040 (5)	−0.004 (3)	−0.005 (3)	−0.011 (3)
C11B	0.024 (3)	0.025 (4)	0.062 (6)	−0.003 (3)	0.002 (4)	0.001 (4)
C12B	0.028 (3)	0.024 (3)	0.043 (5)	−0.001 (3)	−0.002 (3)	0.003 (3)
C13B	0.028 (3)	0.018 (3)	0.043 (5)	0.004 (3)	0.005 (3)	0.003 (3)
O1C	0.028 (2)	0.019 (2)	0.028 (3)	0.0025 (18)	−0.004 (2)	0.001 (2)
O2C	0.031 (3)	0.021 (2)	0.039 (3)	0.004 (2)	0.000 (2)	−0.002 (2)
N1C	0.028 (3)	0.026 (3)	0.030 (3)	−0.003 (2)	0.004 (3)	0.002 (3)
N2C	0.037 (3)	0.031 (3)	0.030 (4)	0.001 (3)	0.008 (3)	0.001 (3)
N3C	0.025 (3)	0.028 (3)	0.028 (3)	−0.003 (2)	0.003 (2)	0.001 (2)
C1C	0.021 (3)	0.025 (3)	0.031 (4)	−0.002 (2)	−0.002 (3)	0.005 (3)
C2C	0.025 (3)	0.025 (3)	0.040 (5)	−0.001 (3)	0.001 (3)	0.009 (3)
C3C	0.029 (4)	0.027 (4)	0.054 (5)	0.003 (3)	−0.001 (3)	0.008 (4)
C4C	0.038 (4)	0.035 (4)	0.043 (5)	0.000 (3)	−0.009 (4)	0.018 (4)
C5C	0.037 (4)	0.039 (4)	0.033 (4)	−0.004 (3)	−0.011 (3)	0.012 (3)
C6C	0.028 (3)	0.025 (3)	0.034 (4)	−0.007 (3)	−0.004 (3)	0.005 (3)
C7C	0.042 (4)	0.027 (4)	0.055 (6)	0.006 (3)	−0.002 (4)	−0.010 (4)
C8C	0.032 (4)	0.037 (4)	0.031 (4)	−0.010 (3)	−0.004 (3)	0.012 (3)
C9C	0.028 (3)	0.031 (4)	0.035 (5)	−0.004 (3)	0.005 (3)	0.004 (3)
C10C	0.044 (4)	0.042 (4)	0.031 (4)	0.010 (4)	0.016 (4)	0.000 (3)
C11C	0.039 (4)	0.047 (5)	0.053 (6)	0.012 (4)	0.015 (4)	−0.004 (4)
C12C	0.037 (4)	0.035 (4)	0.043 (5)	0.007 (3)	0.012 (4)	0.003 (4)
C13C	0.033 (4)	0.030 (4)	0.032 (4)	0.005 (3)	0.004 (3)	0.006 (3)
O1D	0.029 (2)	0.025 (2)	0.028 (3)	0.001 (2)	−0.005 (2)	0.002 (2)
O2D	0.030 (2)	0.032 (3)	0.038 (3)	−0.002 (2)	−0.002 (2)	0.006 (2)
N1D	0.038 (3)	0.028 (3)	0.030 (4)	0.004 (3)	0.006 (3)	0.006 (3)
N2D	0.037 (4)	0.043 (4)	0.027 (4)	0.000 (3)	0.003 (3)	0.009 (3)
N3D	0.039 (3)	0.018 (3)	0.030 (3)	0.004 (2)	0.004 (3)	0.001 (2)
C1D	0.030 (3)	0.021 (3)	0.032 (4)	0.008 (3)	−0.001 (3)	0.000 (3)
C2D	0.033 (4)	0.018 (3)	0.032 (4)	0.005 (3)	0.000 (3)	0.004 (3)
C3D	0.030 (3)	0.028 (3)	0.042 (5)	0.003 (3)	−0.005 (3)	−0.003 (3)
C4D	0.030 (4)	0.041 (4)	0.043 (5)	0.006 (3)	−0.009 (3)	−0.005 (4)
C5D	0.039 (4)	0.034 (4)	0.036 (5)	0.006 (3)	−0.013 (3)	0.002 (3)
C6D	0.036 (4)	0.025 (3)	0.034 (4)	0.005 (3)	−0.005 (3)	−0.002 (3)
C7D	0.045 (5)	0.037 (4)	0.053 (6)	−0.008 (4)	0.006 (4)	0.015 (4)
C8D	0.037 (4)	0.031 (4)	0.030 (4)	0.009 (3)	0.000 (3)	0.005 (3)
C9D	0.042 (4)	0.024 (3)	0.034 (4)	0.009 (3)	0.012 (3)	0.009 (3)
C10D	0.044 (5)	0.036 (4)	0.039 (5)	0.003 (4)	0.017 (4)	0.012 (4)
C11D	0.049 (5)	0.032 (4)	0.058 (6)	0.004 (4)	0.022 (4)	0.008 (4)
C12D	0.037 (4)	0.024 (4)	0.066 (6)	−0.001 (3)	0.007 (4)	−0.007 (4)
C13D	0.033 (4)	0.022 (3)	0.047 (5)	0.001 (3)	0.005 (4)	−0.004 (3)
Cl1	0.0321 (9)	0.0463 (10)	0.0294 (10)	0.0015 (8)	0.0001 (7)	0.0039 (8)
O11	0.037 (3)	0.063 (4)	0.058 (4)	−0.010 (3)	−0.002 (3)	0.007 (3)
O12	0.080 (5)	0.043 (3)	0.051 (4)	−0.009 (3)	−0.002 (4)	0.002 (3)
O13	0.091 (5)	0.081 (5)	0.040 (4)	0.010 (4)	0.022 (4)	−0.001 (4)
O14	0.039 (4)	0.099 (6)	0.076 (5)	0.015 (4)	−0.010 (3)	0.025 (4)
Cl2	0.0553 (12)	0.0398 (11)	0.0434 (13)	0.0063 (9)	0.0047 (10)	0.0041 (9)
O21	0.054 (12)	0.044 (9)	0.034 (9)	0.021 (8)	−0.004 (7)	−0.010 (7)
O22	0.114 (18)	0.054 (10)	0.049 (11)	0.039 (11)	−0.045 (11)	−0.015 (8)
O23	0.064 (10)	0.044 (9)	0.059 (11)	0.026 (7)	0.025 (8)	0.018 (7)
O24	0.066 (11)	0.115 (17)	0.110 (18)	−0.021 (11)	0.008 (10)	0.031 (14)
O21A	0.057 (15)	0.053 (16)	0.049 (16)	−0.011 (11)	−0.001 (12)	0.012 (13)
O22A	0.088 (18)	0.038 (10)	0.032 (10)	0.018 (10)	0.000 (11)	0.001 (7)
O23A	0.064 (13)	0.12 (2)	0.062 (15)	0.035 (15)	−0.011 (11)	0.040 (16)
O24A	0.15 (2)	0.074 (16)	0.056 (14)	−0.063 (16)	−0.006 (15)	−0.003 (11)
Cl3	0.076 (2)	0.126 (3)	0.067 (2)	−0.032 (2)	−0.0087 (16)	0.013 (2)
O31	0.114 (8)	0.178 (10)	0.139 (9)	−0.060 (8)	0.030 (7)	−0.013 (8)
O32	0.078 (7)	0.074 (7)	0.040 (6)	−0.002 (6)	−0.023 (6)	−0.005 (6)
O33	0.066 (7)	0.085 (8)	0.073 (8)	0.021 (7)	−0.024 (7)	−0.008 (7)
O34	0.080 (9)	0.085 (9)	0.062 (8)	−0.005 (8)	−0.043 (7)	−0.002 (7)
O32A	0.056 (7)	0.076 (8)	0.040 (7)	−0.010 (6)	−0.003 (6)	0.017 (6)
O33A	0.097 (8)	0.086 (8)	0.055 (7)	−0.004 (7)	−0.051 (6)	0.001 (6)
O34A	0.079 (10)	0.080 (8)	0.045 (7)	−0.005 (8)	−0.028 (7)	0.007 (6)
Cl4	0.0608 (15)	0.081 (2)	0.074 (2)	0.0103 (14)	0.0146 (14)	0.0152 (16)
O41	0.096 (8)	0.088 (6)	0.046 (6)	−0.016 (6)	−0.020 (5)	0.040 (5)
O42	0.087 (7)	0.044 (5)	0.060 (6)	−0.010 (5)	0.012 (5)	0.009 (4)
O43	0.101 (8)	0.068 (6)	0.108 (9)	0.017 (6)	0.031 (7)	0.010 (6)
O44	0.103 (8)	0.112 (9)	0.046 (6)	−0.010 (7)	−0.025 (6)	0.015 (6)
O41A	0.10 (2)	0.13 (2)	0.07 (2)	−0.014 (18)	0.021 (18)	0.032 (17)
O42A	0.118 (14)	0.098 (14)	0.103 (15)	0.008 (14)	−0.012 (14)	0.010 (13)
O43A	0.067 (15)	0.050 (14)	0.066 (15)	0.016 (13)	0.012 (14)	0.021 (12)
O44A	0.069 (15)	0.098 (16)	0.050 (14)	−0.005 (14)	−0.019 (13)	−0.014 (14)
N1S	0.091 (7)	0.084 (7)	0.050 (6)	−0.039 (6)	−0.019 (5)	0.029 (5)
C11S	0.060 (6)	0.035 (5)	0.058 (7)	−0.003 (4)	−0.017 (5)	−0.001 (4)
C12S	0.112 (10)	0.067 (7)	0.033 (6)	0.025 (7)	−0.003 (6)	−0.010 (5)
O1W	0.046 (7)	0.043 (7)	0.031 (7)	0.002 (5)	−0.003 (5)	0.001 (5)
O1WA	0.14 (2)	0.102 (17)	0.051 (12)	−0.052 (15)	0.027 (11)	−0.034 (11)
O2W	0.063 (4)	0.059 (4)	0.042 (4)	0.012 (4)	0.004 (3)	−0.012 (3)

### Geometric parameters (Å, º)

**Table:**

Ni1—N1A	1.932 (7)	C1C—C6C	1.406 (11)
Ni1—O1A	1.960 (5)	C1C—C2C	1.410 (10)
Ni1—N3A	1.980 (6)	C2C—C3C	1.386 (10)
Ni1—O1B	1.998 (5)	C3C—C4C	1.393 (12)
Ni1—O2B	2.276 (4)	C3C—H3CA	0.9500
Ni2—N1B	1.935 (6)	C4C—C5C	1.363 (12)
Ni2—O1B	1.962 (4)	C4C—H4CA	0.9500
Ni2—N3B	1.971 (6)	C5C—C6C	1.401 (10)
Ni2—O1C	1.997 (5)	C5C—H5CA	0.9500
Ni2—O2C	2.257 (5)	C6C—C8C	1.434 (11)
Ni3—N1C	1.932 (7)	C7C—H7CA	0.9800
Ni3—O1C	1.965 (5)	C7C—H7CB	0.9800
Ni3—N3C	1.975 (6)	C7C—H7CC	0.9800
Ni3—O1D	1.993 (5)	C8C—H8CA	0.9500
Ni3—O2D	2.264 (5)	C9C—C10C	1.409 (11)
Ni4—N1D	1.948 (7)	C10C—C11C	1.355 (13)
Ni4—O1D	1.950 (5)	C10C—H10C	0.9500
Ni4—N3D	1.969 (6)	C11C—C12C	1.397 (13)
Ni4—O1A	1.993 (5)	C11C—H11C	0.9500
Ni4—O2A	2.283 (5)	C12C—C13C	1.375 (10)
O1A—C1A	1.365 (8)	C12C—H12C	0.9500
O2A—C2A	1.378 (10)	C13C—H13C	0.9500
O2A—C7A	1.439 (8)	O1D—C1D	1.360 (9)
N1A—C8A	1.305 (9)	O2D—C2D	1.387 (9)
N1A—N2A	1.377 (9)	O2D—C7D	1.429 (9)
N2A—C9A	1.364 (10)	N1D—C8D	1.300 (10)
N2A—H2AA	0.8800	N1D—N2D	1.362 (9)
N3A—C9A	1.330 (9)	N2D—C9D	1.355 (11)
N3A—C13A	1.356 (9)	N2D—H2DA	0.8800
C1A—C6A	1.396 (11)	N3D—C9D	1.341 (10)
C1A—C2A	1.414 (10)	N3D—C13D	1.351 (10)
C2A—C3A	1.379 (10)	C1D—C2D	1.403 (10)
C3A—C4A	1.370 (13)	C1D—C6D	1.410 (11)
C3A—H3AA	0.9500	C2D—C3D	1.383 (10)
C4A—C5A	1.378 (13)	C3D—C4D	1.373 (12)
C4A—H4AA	0.9500	C3D—H3DA	0.9500
C5A—C6A	1.403 (10)	C4D—C5D	1.368 (12)
C5A—H5AA	0.9500	C4D—H4DA	0.9500
C6A—C8A	1.441 (11)	C5D—C6D	1.416 (11)
C7A—H7AA	0.9800	C5D—H5DA	0.9500
C7A—H7AB	0.9800	C6D—C8D	1.433 (11)
C7A—H7AC	0.9800	C7D—H7DA	0.9800
C8A—H8AA	0.9500	C7D—H7DB	0.9800
C9A—C10A	1.411 (10)	C7D—H7DC	0.9800
C10A—C11A	1.330 (12)	C8D—H8DA	0.9500
C10A—H10A	0.9500	C9D—C10D	1.401 (11)
C11A—C12A	1.408 (12)	C10D—C11D	1.390 (13)
C11A—H11A	0.9500	C10D—H10D	0.9500
C12A—C13A	1.384 (10)	C11D—C12D	1.381 (14)
C12A—H12A	0.9500	C11D—H11D	0.9500
C13A—H13A	0.9500	C12D—C13D	1.380 (11)
O1B—C1B	1.367 (8)	C12D—H12D	0.9500
O2B—C2B	1.380 (8)	C13D—H13D	0.9500
O2B—C7B	1.435 (8)	Cl1—O14	1.409 (6)
N1B—C8B	1.292 (9)	Cl1—O13	1.413 (7)
N1B—N2B	1.377 (8)	Cl1—O12	1.424 (6)
N2B—C9B	1.353 (10)	Cl1—O11	1.429 (6)
N2B—H2BA	0.8800	Cl2—O24A	1.378 (18)
N3B—C9B	1.351 (10)	Cl2—O21A	1.397 (18)
N3B—C13B	1.360 (9)	Cl2—O23	1.399 (14)
C1B—C2B	1.402 (9)	Cl2—O21	1.405 (15)
C1B—C6B	1.404 (10)	Cl2—O22	1.430 (14)
C2B—C3B	1.387 (10)	Cl2—O22A	1.437 (17)
C3B—C4B	1.398 (10)	Cl2—O24	1.438 (14)
C3B—H3BA	0.9500	Cl2—O23A	1.449 (17)
C4B—C5B	1.364 (10)	Cl3—O33A	1.248 (12)
C4B—H4BA	0.9500	Cl3—O34A	1.326 (15)
C5B—C6B	1.414 (10)	Cl3—O32	1.336 (12)
C5B—H5BA	0.9500	Cl3—O34	1.377 (15)
C6B—C8B	1.453 (10)	Cl3—O31	1.448 (9)
C7B—H7BA	0.9800	Cl3—O33	1.517 (12)
C7B—H7BB	0.9800	Cl3—O32A	1.677 (14)
C7B—H7BC	0.9800	Cl4—O41	1.363 (9)
C8B—H8BA	0.9500	Cl4—O44	1.368 (9)
C9B—C10B	1.408 (10)	Cl4—O44A	1.38 (2)
C10B—C11B	1.356 (12)	Cl4—O41A	1.40 (2)
C10B—H10B	0.9500	Cl4—O43A	1.40 (2)
C11B—C12B	1.411 (12)	Cl4—O42	1.420 (9)
C11B—H11B	0.9500	Cl4—O43	1.457 (10)
C12B—C13B	1.360 (10)	Cl4—O42A	1.47 (2)
C12B—H12B	0.9500	N1S—C11S	1.117 (13)
C13B—H13B	0.9500	C11S—C12S	1.462 (15)
O1C—C1C	1.355 (8)	C12S—H12E	0.9800
O2C—C2C	1.368 (9)	C12S—H12F	0.9800
O2C—C7C	1.439 (9)	C12S—H12G	0.9800
N1C—C8C	1.290 (9)	O1W—H1W1	0.80 (3)
N1C—N2C	1.381 (9)	O1W—H1W2	0.82 (3)
N2C—C9C	1.371 (10)	O1WA—H1W3	0.82 (3)
N2C—H2CA	0.8800	O1WA—H1W4	0.82 (3)
N3C—C13C	1.341 (9)	O2W—H2W1	0.84 (3)
N3C—C9C	1.347 (10)	O2W—H2W2	0.83 (3)
			
N1A—Ni1—O1A	92.2 (2)	C8C—N1C—N2C	118.7 (7)
N1A—Ni1—N3A	81.6 (2)	C8C—N1C—Ni3	128.3 (6)
O1A—Ni1—N3A	173.5 (2)	N2C—N1C—Ni3	112.8 (5)
N1A—Ni1—O1B	171.2 (2)	C9C—N2C—N1C	115.9 (7)
O1A—Ni1—O1B	87.1 (2)	C9C—N2C—H2CA	122.1
N3A—Ni1—O1B	98.8 (2)	N1C—N2C—H2CA	122.1
N1A—Ni1—O2B	113.4 (2)	C13C—N3C—C9C	118.6 (6)
O1A—Ni1—O2B	98.83 (19)	C13C—N3C—Ni3	129.0 (5)
N3A—Ni1—O2B	85.3 (2)	C9C—N3C—Ni3	112.4 (5)
O1B—Ni1—O2B	75.39 (17)	O1C—C1C—C6C	123.0 (6)
N1B—Ni2—O1B	91.3 (2)	O1C—C1C—C2C	118.7 (7)
N1B—Ni2—N3B	82.0 (2)	C6C—C1C—C2C	118.3 (6)
O1B—Ni2—N3B	171.8 (2)	O2C—C2C—C3C	124.2 (7)
N1B—Ni2—O1C	172.1 (2)	O2C—C2C—C1C	114.4 (6)
O1B—Ni2—O1C	88.8 (2)	C3C—C2C—C1C	121.3 (8)
N3B—Ni2—O1C	97.1 (2)	C2C—C3C—C4C	119.6 (7)
N1B—Ni2—O2C	111.7 (2)	C2C—C3C—H3CA	120.2
O1B—Ni2—O2C	97.69 (18)	C4C—C3C—H3CA	120.2
N3B—Ni2—O2C	89.2 (2)	C5C—C4C—C3C	119.8 (7)
O1C—Ni2—O2C	76.02 (19)	C5C—C4C—H4CA	120.1
N1C—Ni3—O1C	91.2 (2)	C3C—C4C—H4CA	120.1
N1C—Ni3—N3C	82.4 (3)	C4C—C5C—C6C	122.0 (8)
O1C—Ni3—N3C	172.3 (2)	C4C—C5C—H5CA	119.0
N1C—Ni3—O1D	172.1 (2)	C6C—C5C—H5CA	119.0
O1C—Ni3—O1D	88.1 (2)	C5C—C6C—C1C	119.0 (7)
N3C—Ni3—O1D	97.7 (2)	C5C—C6C—C8C	117.2 (8)
N1C—Ni3—O2D	111.5 (2)	C1C—C6C—C8C	123.8 (7)
O1C—Ni3—O2D	99.81 (19)	O2C—C7C—H7CA	109.5
N3C—Ni3—O2D	86.5 (2)	O2C—C7C—H7CB	109.5
O1D—Ni3—O2D	76.3 (2)	H7CA—C7C—H7CB	109.5
N1D—Ni4—O1D	89.9 (2)	O2C—C7C—H7CC	109.5
N1D—Ni4—N3D	81.9 (3)	H7CA—C7C—H7CC	109.5
O1D—Ni4—N3D	170.6 (2)	H7CB—C7C—H7CC	109.5
N1D—Ni4—O1A	169.1 (2)	N1C—C8C—C6C	125.2 (7)
O1D—Ni4—O1A	89.5 (2)	N1C—C8C—H8CA	117.4
N3D—Ni4—O1A	97.8 (2)	C6C—C8C—H8CA	117.4
N1D—Ni4—O2A	116.3 (2)	N3C—C9C—N2C	116.4 (7)
O1D—Ni4—O2A	100.3 (2)	N3C—C9C—C10C	122.5 (7)
N3D—Ni4—O2A	87.4 (2)	N2C—C9C—C10C	121.1 (7)
O1A—Ni4—O2A	74.5 (2)	C11C—C10C—C9C	117.5 (8)
C1A—O1A—Ni1	125.5 (4)	C11C—C10C—H10C	121.3
C1A—O1A—Ni4	121.3 (4)	C9C—C10C—H10C	121.3
Ni1—O1A—Ni4	112.2 (2)	C10C—C11C—C12C	120.5 (8)
C2A—O2A—C7A	116.2 (6)	C10C—C11C—H11C	119.7
C2A—O2A—Ni4	113.0 (4)	C12C—C11C—H11C	119.7
C7A—O2A—Ni4	129.4 (5)	C13C—C12C—C11C	118.8 (8)
C8A—N1A—N2A	118.3 (7)	C13C—C12C—H12C	120.6
C8A—N1A—Ni1	128.8 (5)	C11C—C12C—H12C	120.6
N2A—N1A—Ni1	112.9 (5)	N3C—C13C—C12C	122.0 (8)
C9A—N2A—N1A	115.8 (6)	N3C—C13C—H13C	119.0
C9A—N2A—H2AA	122.1	C12C—C13C—H13C	119.0
N1A—N2A—H2AA	122.1	C1D—O1D—Ni4	127.6 (5)
C9A—N3A—C13A	118.5 (6)	C1D—O1D—Ni3	118.5 (4)
C9A—N3A—Ni1	113.1 (5)	Ni4—O1D—Ni3	112.3 (2)
C13A—N3A—Ni1	128.2 (5)	C2D—O2D—C7D	117.2 (6)
O1A—C1A—C6A	123.8 (6)	C2D—O2D—Ni3	111.3 (4)
O1A—C1A—C2A	117.9 (7)	C7D—O2D—Ni3	126.9 (5)
C6A—C1A—C2A	118.3 (6)	C8D—N1D—N2D	118.1 (7)
O2A—C2A—C3A	125.7 (7)	C8D—N1D—Ni4	129.7 (5)
O2A—C2A—C1A	113.3 (6)	N2D—N1D—Ni4	112.2 (5)
C3A—C2A—C1A	120.8 (8)	C9D—N2D—N1D	116.1 (7)
C4A—C3A—C2A	120.2 (8)	C9D—N2D—H2DA	121.9
C4A—C3A—H3AA	119.9	N1D—N2D—H2DA	121.9
C2A—C3A—H3AA	119.9	C9D—N3D—C13D	120.3 (7)
C3A—C4A—C5A	120.5 (8)	C9D—N3D—Ni4	112.0 (5)
C3A—C4A—H4AA	119.7	C13D—N3D—Ni4	127.7 (6)
C5A—C4A—H4AA	119.7	O1D—C1D—C2D	119.7 (7)
C4A—C5A—C6A	120.3 (9)	O1D—C1D—C6D	122.9 (6)
C4A—C5A—H5AA	119.8	C2D—C1D—C6D	117.2 (7)
C6A—C5A—H5AA	119.8	C3D—C2D—O2D	124.0 (6)
C1A—C6A—C5A	119.8 (7)	C3D—C2D—C1D	122.2 (7)
C1A—C6A—C8A	124.7 (6)	O2D—C2D—C1D	113.8 (6)
C5A—C6A—C8A	115.5 (7)	C4D—C3D—C2D	119.8 (7)
O2A—C7A—H7AA	109.5	C4D—C3D—H3DA	120.1
O2A—C7A—H7AB	109.5	C2D—C3D—H3DA	120.1
H7AA—C7A—H7AB	109.5	C5D—C4D—C3D	120.5 (7)
O2A—C7A—H7AC	109.5	C5D—C4D—H4DA	119.7
H7AA—C7A—H7AC	109.5	C3D—C4D—H4DA	119.7
H7AB—C7A—H7AC	109.5	C4D—C5D—C6D	120.5 (8)
N1A—C8A—C6A	123.6 (7)	C4D—C5D—H5DA	119.7
N1A—C8A—H8AA	118.2	C6D—C5D—H5DA	119.7
C6A—C8A—H8AA	118.2	C1D—C6D—C5D	119.8 (7)
N3A—C9A—N2A	116.2 (6)	C1D—C6D—C8D	123.8 (7)
N3A—C9A—C10A	122.2 (7)	C5D—C6D—C8D	116.4 (7)
N2A—C9A—C10A	121.6 (7)	O2D—C7D—H7DA	109.5
C11A—C10A—C9A	118.8 (8)	O2D—C7D—H7DB	109.5
C11A—C10A—H10A	120.6	H7DA—C7D—H7DB	109.5
C9A—C10A—H10A	120.6	O2D—C7D—H7DC	109.5
C10A—C11A—C12A	120.7 (7)	H7DA—C7D—H7DC	109.5
C10A—C11A—H11A	119.6	H7DB—C7D—H7DC	109.5
C12A—C11A—H11A	119.6	N1D—C8D—C6D	123.7 (7)
C13A—C12A—C11A	117.5 (7)	N1D—C8D—H8DA	118.2
C13A—C12A—H12A	121.2	C6D—C8D—H8DA	118.2
C11A—C12A—H12A	121.2	N3D—C9D—N2D	117.1 (7)
N3A—C13A—C12A	122.2 (7)	N3D—C9D—C10D	121.9 (8)
N3A—C13A—H13A	118.9	N2D—C9D—C10D	121.0 (8)
C12A—C13A—H13A	118.9	C11D—C10D—C9D	117.1 (8)
C1B—O1B—Ni2	125.4 (4)	C11D—C10D—H10D	121.5
C1B—O1B—Ni1	118.9 (4)	C9D—C10D—H10D	121.5
Ni2—O1B—Ni1	111.5 (2)	C12D—C11D—C10D	120.7 (8)
C2B—O2B—C7B	117.9 (5)	C12D—C11D—H11D	119.6
C2B—O2B—Ni1	111.1 (4)	C10D—C11D—H11D	119.6
C7B—O2B—Ni1	125.4 (4)	C13D—C12D—C11D	119.1 (8)
C8B—N1B—N2B	118.7 (6)	C13D—C12D—H12D	120.4
C8B—N1B—Ni2	129.1 (5)	C11D—C12D—H12D	120.4
N2B—N1B—Ni2	112.1 (4)	N3D—C13D—C12D	120.8 (9)
C9B—N2B—N1B	116.7 (6)	N3D—C13D—H13D	119.6
C9B—N2B—H2BA	121.7	C12D—C13D—H13D	119.6
N1B—N2B—H2BA	121.7	O14—Cl1—O13	111.7 (5)
C9B—N3B—C13B	119.4 (6)	O14—Cl1—O12	108.8 (5)
C9B—N3B—Ni2	112.6 (5)	O13—Cl1—O12	109.5 (4)
C13B—N3B—Ni2	128.0 (5)	O14—Cl1—O11	109.3 (4)
O1B—C1B—C2B	118.5 (6)	O13—Cl1—O11	109.1 (5)
O1B—C1B—C6B	122.7 (6)	O12—Cl1—O11	108.3 (4)
C2B—C1B—C6B	118.8 (6)	O24A—Cl2—O21A	112.5 (17)
O2B—C2B—C3B	123.8 (6)	O23—Cl2—O21	111.8 (11)
O2B—C2B—C1B	114.3 (6)	O23—Cl2—O22	107.8 (11)
C3B—C2B—C1B	121.8 (7)	O21—Cl2—O22	108.6 (13)
C2B—C3B—C4B	118.7 (7)	O24A—Cl2—O22A	111.1 (14)
C2B—C3B—H3BA	120.6	O21A—Cl2—O22A	108.6 (16)
C4B—C3B—H3BA	120.6	O23—Cl2—O24	112.1 (12)
C5B—C4B—C3B	120.7 (7)	O21—Cl2—O24	106.6 (13)
C5B—C4B—H4BA	119.7	O22—Cl2—O24	109.9 (12)
C3B—C4B—H4BA	119.7	O24A—Cl2—O23A	111.2 (15)
C4B—C5B—C6B	121.2 (7)	O21A—Cl2—O23A	109.4 (17)
C4B—C5B—H5BA	119.4	O22A—Cl2—O23A	103.7 (15)
C6B—C5B—H5BA	119.4	O33A—Cl3—O34A	131.5 (15)
C1B—C6B—C5B	118.8 (6)	O32—Cl3—O34	117.6 (13)
C1B—C6B—C8B	125.1 (6)	O33A—Cl3—O31	109.2 (10)
C5B—C6B—C8B	116.1 (6)	O34A—Cl3—O31	110.4 (13)
O2B—C7B—H7BA	109.5	O32—Cl3—O31	111.5 (9)
O2B—C7B—H7BB	109.5	O34—Cl3—O31	103.8 (12)
H7BA—C7B—H7BB	109.5	O32—Cl3—O33	108.8 (9)
O2B—C7B—H7BC	109.5	O34—Cl3—O33	109.1 (13)
H7BA—C7B—H7BC	109.5	O31—Cl3—O33	105.3 (8)
H7BB—C7B—H7BC	109.5	O33A—Cl3—O32A	100.3 (10)
N1B—C8B—C6B	122.9 (7)	O34A—Cl3—O32A	102.7 (13)
N1B—C8B—H8BA	118.6	O31—Cl3—O32A	95.4 (7)
C6B—C8B—H8BA	118.6	O41—Cl4—O44	120.0 (7)
N3B—C9B—N2B	115.9 (6)	O44A—Cl4—O41A	114 (2)
N3B—C9B—C10B	121.2 (7)	O44A—Cl4—O43A	111.8 (18)
N2B—C9B—C10B	122.9 (7)	O41A—Cl4—O43A	111 (2)
C11B—C10B—C9B	118.5 (8)	O41—Cl4—O42	108.1 (7)
C11B—C10B—H10B	120.7	O44—Cl4—O42	109.7 (7)
C9B—C10B—H10B	120.7	O41—Cl4—O43	101.2 (7)
C10B—C11B—C12B	120.2 (7)	O44—Cl4—O43	104.3 (8)
C10B—C11B—H11B	119.9	O42—Cl4—O43	113.3 (7)
C12B—C11B—H11B	119.9	O44A—Cl4—O42A	110.2 (17)
C13B—C12B—C11B	118.8 (7)	O41A—Cl4—O42A	104.0 (18)
C13B—C12B—H12B	120.6	O43A—Cl4—O42A	105.6 (18)
C11B—C12B—H12B	120.6	N1S—C11S—C12S	177.7 (12)
C12B—C13B—N3B	121.7 (8)	C11S—C12S—H12E	109.5
C12B—C13B—H13B	119.1	C11S—C12S—H12F	109.5
N3B—C13B—H13B	119.1	H12E—C12S—H12F	109.5
C1C—O1C—Ni3	126.9 (5)	C11S—C12S—H12G	109.5
C1C—O1C—Ni2	118.8 (4)	H12E—C12S—H12G	109.5
Ni3—O1C—Ni2	112.6 (2)	H12F—C12S—H12G	109.5
C2C—O2C—C7C	118.5 (6)	H1W1—O1W—H1W2	107 (5)
C2C—O2C—Ni2	111.7 (4)	H1W3—O1WA—H1W4	108 (5)
C7C—O2C—Ni2	127.8 (5)	H2W1—O2W—H2W2	102 (4)
			
C8A—N1A—N2A—C9A	−174.9 (6)	C8C—N1C—N2C—C9C	−176.2 (6)
Ni1—N1A—N2A—C9A	6.2 (7)	Ni3—N1C—N2C—C9C	−0.8 (7)
Ni1—O1A—C1A—C6A	−14.8 (9)	Ni3—O1C—C1C—C6C	−12.1 (9)
Ni4—O1A—C1A—C6A	177.7 (5)	Ni2—O1C—C1C—C6C	−175.5 (5)
Ni1—O1A—C1A—C2A	167.0 (4)	Ni3—O1C—C1C—C2C	169.2 (5)
Ni4—O1A—C1A—C2A	−0.5 (8)	Ni2—O1C—C1C—C2C	5.8 (8)
C7A—O2A—C2A—C3A	17.2 (10)	C7C—O2C—C2C—C3C	11.8 (10)
Ni4—O2A—C2A—C3A	−175.2 (6)	Ni2—O2C—C2C—C3C	176.9 (6)
C7A—O2A—C2A—C1A	−166.9 (6)	C7C—O2C—C2C—C1C	−169.2 (6)
Ni4—O2A—C2A—C1A	0.8 (7)	Ni2—O2C—C2C—C1C	−4.1 (7)
O1A—C1A—C2A—O2A	−0.2 (8)	O1C—C1C—C2C—O2C	−0.5 (9)
C6A—C1A—C2A—O2A	−178.6 (6)	C6C—C1C—C2C—O2C	−179.3 (6)
O1A—C1A—C2A—C3A	175.9 (6)	O1C—C1C—C2C—C3C	178.5 (6)
C6A—C1A—C2A—C3A	−2.4 (10)	C6C—C1C—C2C—C3C	−0.3 (10)
O2A—C2A—C3A—C4A	176.9 (7)	O2C—C2C—C3C—C4C	178.5 (7)
C1A—C2A—C3A—C4A	1.2 (11)	C1C—C2C—C3C—C4C	−0.5 (11)
C2A—C3A—C4A—C5A	0.1 (12)	C2C—C3C—C4C—C5C	0.6 (12)
C3A—C4A—C5A—C6A	−0.2 (12)	C3C—C4C—C5C—C6C	0.1 (12)
O1A—C1A—C6A—C5A	−175.9 (6)	C4C—C5C—C6C—C1C	−0.8 (11)
C2A—C1A—C6A—C5A	2.3 (10)	C4C—C5C—C6C—C8C	179.5 (7)
O1A—C1A—C6A—C8A	5.5 (11)	O1C—C1C—C6C—C5C	−177.8 (6)
C2A—C1A—C6A—C8A	−176.2 (6)	C2C—C1C—C6C—C5C	0.9 (10)
C4A—C5A—C6A—C1A	−1.1 (11)	O1C—C1C—C6C—C8C	1.8 (10)
C4A—C5A—C6A—C8A	177.6 (7)	C2C—C1C—C6C—C8C	−179.5 (6)
N2A—N1A—C8A—C6A	178.6 (6)	N2C—N1C—C8C—C6C	179.8 (6)
Ni1—N1A—C8A—C6A	−2.7 (11)	Ni3—N1C—C8C—C6C	5.2 (10)
C1A—C6A—C8A—N1A	3.8 (11)	C5C—C6C—C8C—N1C	−178.4 (7)
C5A—C6A—C8A—N1A	−174.8 (7)	C1C—C6C—C8C—N1C	1.9 (11)
C13A—N3A—C9A—N2A	178.7 (6)	C13C—N3C—C9C—N2C	175.7 (6)
Ni1—N3A—C9A—N2A	2.9 (7)	Ni3—N3C—C9C—N2C	−4.9 (8)
C13A—N3A—C9A—C10A	−2.2 (10)	C13C—N3C—C9C—C10C	−2.6 (10)
Ni1—N3A—C9A—C10A	−178.0 (5)	Ni3—N3C—C9C—C10C	176.8 (6)
N1A—N2A—C9A—N3A	−6.0 (9)	N1C—N2C—C9C—N3C	3.9 (9)
N1A—N2A—C9A—C10A	174.9 (6)	N1C—N2C—C9C—C10C	−177.8 (7)
N3A—C9A—C10A—C11A	0.4 (11)	N3C—C9C—C10C—C11C	1.4 (12)
N2A—C9A—C10A—C11A	179.4 (7)	N2C—C9C—C10C—C11C	−176.8 (7)
C9A—C10A—C11A—C12A	1.9 (11)	C9C—C10C—C11C—C12C	0.4 (13)
C10A—C11A—C12A—C13A	−2.2 (11)	C10C—C11C—C12C—C13C	−1.0 (13)
C9A—N3A—C13A—C12A	1.8 (10)	C9C—N3C—C13C—C12C	2.0 (10)
Ni1—N3A—C13A—C12A	176.9 (5)	Ni3—N3C—C13C—C12C	−177.3 (6)
C11A—C12A—C13A—N3A	0.4 (11)	C11C—C12C—C13C—N3C	−0.3 (12)
C8B—N1B—N2B—C9B	168.2 (6)	C8D—N1D—N2D—C9D	172.1 (6)
Ni2—N1B—N2B—C9B	−8.9 (7)	Ni4—N1D—N2D—C9D	−7.5 (8)
Ni2—O1B—C1B—C2B	−165.5 (4)	Ni4—O1D—C1D—C2D	−169.2 (4)
Ni1—O1B—C1B—C2B	−10.6 (7)	Ni3—O1D—C1D—C2D	−5.0 (8)
Ni2—O1B—C1B—C6B	16.7 (9)	Ni4—O1D—C1D—C6D	14.1 (9)
Ni1—O1B—C1B—C6B	171.7 (5)	Ni3—O1D—C1D—C6D	178.3 (5)
C7B—O2B—C2B—C3B	−16.1 (10)	C7D—O2D—C2D—C3D	−20.3 (10)
Ni1—O2B—C2B—C3B	−171.2 (5)	Ni3—O2D—C2D—C3D	−178.1 (5)
C7B—O2B—C2B—C1B	164.7 (6)	C7D—O2D—C2D—C1D	162.5 (6)
Ni1—O2B—C2B—C1B	9.6 (7)	Ni3—O2D—C2D—C1D	4.7 (7)
O1B—C1B—C2B—O2B	−0.7 (9)	O1D—C1D—C2D—C3D	−177.7 (6)
C6B—C1B—C2B—O2B	177.2 (6)	C6D—C1D—C2D—C3D	−0.8 (9)
O1B—C1B—C2B—C3B	−179.9 (6)	O1D—C1D—C2D—O2D	−0.4 (9)
C6B—C1B—C2B—C3B	−2.1 (10)	C6D—C1D—C2D—O2D	176.5 (6)
O2B—C2B—C3B—C4B	−178.2 (6)	O2D—C2D—C3D—C4D	−177.0 (7)
C1B—C2B—C3B—C4B	1.0 (10)	C1D—C2D—C3D—C4D	0.0 (10)
C2B—C3B—C4B—C5B	1.0 (11)	C2D—C3D—C4D—C5D	1.0 (11)
C3B—C4B—C5B—C6B	−1.9 (11)	C3D—C4D—C5D—C6D	−1.3 (12)
O1B—C1B—C6B—C5B	178.9 (6)	O1D—C1D—C6D—C5D	177.3 (6)
C2B—C1B—C6B—C5B	1.1 (10)	C2D—C1D—C6D—C5D	0.5 (10)
O1B—C1B—C6B—C8B	−0.5 (10)	O1D—C1D—C6D—C8D	0.6 (11)
C2B—C1B—C6B—C8B	−178.3 (6)	C2D—C1D—C6D—C8D	−176.2 (6)
C4B—C5B—C6B—C1B	0.8 (11)	C4D—C5D—C6D—C1D	0.5 (11)
C4B—C5B—C6B—C8B	−179.7 (7)	C4D—C5D—C6D—C8D	177.5 (7)
N2B—N1B—C8B—C6B	177.2 (6)	N2D—N1D—C8D—C6D	177.0 (7)
Ni2—N1B—C8B—C6B	−6.3 (10)	Ni4—N1D—C8D—C6D	−3.5 (11)
C1B—C6B—C8B—N1B	−5.2 (11)	C1D—C6D—C8D—N1D	−6.1 (11)
C5B—C6B—C8B—N1B	175.3 (6)	C5D—C6D—C8D—N1D	177.1 (7)
C13B—N3B—C9B—N2B	−178.2 (6)	C13D—N3D—C9D—N2D	−175.4 (6)
Ni2—N3B—C9B—N2B	2.5 (7)	Ni4—N3D—C9D—N2D	3.8 (8)
C13B—N3B—C9B—C10B	3.5 (10)	C13D—N3D—C9D—C10D	3.8 (10)
Ni2—N3B—C9B—C10B	−175.8 (5)	Ni4—N3D—C9D—C10D	−177.0 (6)
N1B—N2B—C9B—N3B	4.2 (9)	N1D—N2D—C9D—N3D	2.5 (10)
N1B—N2B—C9B—C10B	−177.5 (6)	N1D—N2D—C9D—C10D	−176.8 (7)
N3B—C9B—C10B—C11B	−2.8 (11)	N3D—C9D—C10D—C11D	−3.0 (11)
N2B—C9B—C10B—C11B	179.1 (7)	N2D—C9D—C10D—C11D	176.2 (7)
C9B—C10B—C11B—C12B	−0.2 (11)	C9D—C10D—C11D—C12D	0.4 (12)
C10B—C11B—C12B—C13B	2.4 (11)	C10D—C11D—C12D—C13D	1.2 (12)
C11B—C12B—C13B—N3B	−1.6 (10)	C9D—N3D—C13D—C12D	−2.1 (10)
C9B—N3B—C13B—C12B	−1.3 (9)	Ni4—N3D—C13D—C12D	178.9 (5)
Ni2—N3B—C13B—C12B	177.9 (5)	C11D—C12D—C13D—N3D	−0.4 (11)

### Hydrogen-bond geometry (Å, º)

**Table:**

*D*—H···*A*	*D*—H	H···*A*	*D*···*A*	*D*—H···*A*
C7*B*—H7*BB*···N1*S*^i^	0.98	2.60	3.523 (11)	157
C13*B*—H13*B*···O13	0.95	2.42	3.126 (9)	131
C13*C*—H13*C*···N1*S*	0.95	2.59	3.348 (11)	137
N2*D*—H2*DA*···O2*W*	0.88	1.91	2.720 (9)	152
C7*D*—H7*DC*···O12	0.98	2.56	3.389 (10)	142
C12*S*—H12*G*···O14^ii^	0.98	2.37	3.335 (12)	168
O2*W*—H2*W*1···O11^ii^	0.84 (3)	2.12 (7)	2.795 (8)	138 (9)
O2*W*—H2*W*2···Cl4^ii^	0.83 (3)	2.78 (3)	3.599 (6)	170 (9)

Symmetry codes: (i) *x*+1/2, −*y*+3/2, *z*; (ii) −*x*+1, −*y*+1, *z*−1/2.
